# A Novel Computational Tool for Mining Real-Life Data: Application in the Metastatic Colorectal Cancer Care Setting

**DOI:** 10.1371/journal.pone.0154689

**Published:** 2016-05-04

**Authors:** Nava Siegelmann-Danieli, Ariel Farkash, Itzhak Katzir, Janet Vesterman Landes, Hadas Rotem Rabinovich, Yossef Lomnicky, Boaz Carmeli, Naama Parush-Shear-Yashuv

**Affiliations:** 1 Department of Professional Medicine, Maccabi Healthcare Services, Tel Aviv, Israel; 2 Department of Biomedical Informatics, IBM Research-Haifa, Haifa University Campus, Haifa, Israel; 3 Department of Pharmacology, Maccabi Healthcare Services, Tel Aviv, Israel; Faculté de médecine de Nantes, FRANCE

## Abstract

**Background:**

Randomized clinical trials constitute the gold-standard for evaluating new anti-cancer therapies; however, real-life data are key in complementing clinically useful information. We developed a computational tool for real-life data analysis and applied it to the metastatic colorectal cancer (mCRC) setting. This tool addressed the impact of oncology/non-oncology parameters on treatment patterns and clinical outcomes.

**Methods:**

The developed tool enables extraction of any computerized information including comorbidities and use of drugs (oncological/non-oncological) per individual HMO member. The study in which we evaluated this tool was a retrospective cohort study that included Maccabi Healthcare Services members with mCRC receiving bevacizumab with fluoropyrimidines (FP), FP plus oxaliplatin (FP-O), or FP plus irinotecan (FP-I) in the first-line between 9/2006 and 12/2013.

**Results:**

The analysis included 753 patients of whom 15.4% underwent subsequent metastasectomy (the Surgery group). For the entire cohort, median overall survival (OS) was 20.5 months; in the Surgery group, median duration of bevacizumab-containing therapy (DOT) pre-surgery was 6.1 months; median OS was not reached. In the Non-surgery group, median OS and DOT were 18.7 and 11.4 months, respectively; no significant OS differences were noted between FP-O and FP-I, whereas FP use was associated with shorter OS (12.3 month; *p* <0.002; notably, these patients were older). Patients who received both FP-O- and FP-I-based regimens achieved numerically longer OS vs. those who received only one of these regimens (22.1 [19.9–24.0] vs. 18.9 [15.5–21.9] months). Among patients assessed for wild-type KRAS and treated with subsequent anti-EGFR agent, OS was 25.4 months and 18.7 months for 124 treated vs. 37 non-treated patients (non-significant). Cox analysis (controlling for age and gender) identified several non-oncology parameters associated with poorer clinical outcomes including concurrent use of diuretics and proton-pump inhibitors.

**Conclusions:**

Our tool provided insights that confirmed/complemented information gained from randomized-clinical trials. Prospective tool implementation is warranted.

## Introduction

Prospective randomized clinical trials (RCTs) constitute the gold-standard for evaluation and approval of new anti-cancer therapies; still, they represent experience in selected groups of well-fit patients, with underrepresentation of those with comorbidities, elderly, women, and racial/ethnic minorities [1–4]. Real-life data complement RCT-generated findings with information on post-marketing use, toxicity, interactions with non-oncology factors, and evaluation of various approaches in the absence of head-to-head clinical trials. Longitudinal databases such as those managed by large health maintenance organizations (HMOs) are mostly an untapped source for real-life clinical practice data.

The aim of the current work was to develop an interactive computerized tool that could systematically extract data from various HMO databases and link all the information associated with each individual patient. This system may provide clinicians with insights regarding the optimal treatment algorithms for a specific patient (considering age, gender, comorbidities, and previous/concomitant therapies). It may also help the HMO assess treatment paradigms, search for non-oncological factors affecting outcomes (such as non-cancer regularly-used medications) and evaluate policies. This tool was applied to Maccabi Healthcare Services (MHS), the 2^nd^ largest HMO in Israel, insuring approximately 2 million members, including close to 14,000 new cancer patients annually. Specifically, the tool was applied to colorectal cancer (CRC) patients who received bevacizumab-containing regimen as first-line treatment for metastatic disease. This test case was chosen because the number of patients was expected to be relatively high (CRC is the second most common cancer in women and the third most common cancer in men worldwide) [5]; the follow-up required was expected to be relatively short (RCT data from the initial phase III studies suggest an overall survival [OS] of up to 25 months) [6–10]; and because bevacizumab has been routinely used and reimbursed in this setting in Israel since its coverage under the Israeli National Health Insurance Law was approved in September 2006 and therefore our data is felt to accurately reflect its use. Herein, the tool was used to study the impact of oncology and non-oncology parameters on treatment patterns and clinical outcomes in this setting.

## Methods

### Study Design and patient eligibility

The study was approved by the institutional review board of Maccabi Healthcare Services. Patient information was anonymized and de-identified prior to analysis. This retrospective analysis included all MHS CRC patients who were treated with bevacizumab-containing regimen as first-line therapy in the metastatic setting from September 2006 through 2012. Patients were followed until death or the study cutoff date (December 31, 2013). For patients who were alive at the cutoff date, a minimum of 12 months of follow up from bevacizumab treatment initiation was required for inclusion in the analysis.

### Data source

Individual information for each eligible patient was extracted from the MHS database including demographic information; inclusion in MHS registries for diabetes, hypertension, and cardiovascular diseases; pharmacy records for oncology drugs including the anti-epidermal growth factor receptor (EGFR) agents cetuximab and panitumumab (which were approved by MHS in April 2007 [prior to their reimbursement under the National Health Insurance Law] and their use has been restricted to patients with wild-type (w.t.) KRAS since December 2008); pharmacy records for regular use of non-oncology drugs (defined herein as drugs that were purchased at least 4 out of 12 months starting one year prior to initiation of bevacizumab therapy and until last follow up); hospital claims for surgeries, infusions of chemotherapeutics/biologic agents; and laboratory test results (performed within 6 months prior to initiation of bevacizumab therapy and until last follow up) including blood counts, renal and liver function, lipid panel tests, tumor markers, and KRAS testing. Medical records for patients who discontinued any systemic therapy for at least 15 months, had no records for billing of resection of liver metastases, and were alive at last follow up (n = 44), were reviewed by an oncologist to confirm treatment and health status.

### The computerized tool

The computerized tool includes two tiers: A knowledge-guided tier, which provides insights regarding each patient based on available guidelines, and the descriptive analysis tier which provides insights generated from analysis of the medical records of patients within an organization (MHS in our study). In the current report, only the latter tier was used. The computerized tool retrieves the required data (structured and unstructured) from the HMO database automatically. The unstructured data are retrieved using natural language processing (NLP) techniques [11]. The IBM Advanced Care Insights Platform (ACI) [12] is used to run the Unstructured Information Management Architecture (UIMA) framework [13]. Within ACI, IBM Content Analytic Studio (ICA studio) was used to build a Processing Engine Archive File (PEAR). ACI includes built-in medical dictionaries such as RxNorm, SNOMED CT, ICD-9, ICD-10, LOINC, and HL7, as well as entity mapping (e.g., ICD-9 to ICD-10). For the development of the tool, the ACI was supplemented with a dictionary of chemotherapy drugs and tumor parameters [11, 14].

The descriptive analysis tier applied a formalized approach to create a timeline of treatments (1^st^ line, 2^nd^ line, etc) for each patient, and then used this timeline as an anchor for integrating other clinical data such as surgeries and lab reports. A change in the line of treatment was defined as omitting or replacing a biologic agent or changing the chemotherapy regimen which, in this case, included fluoropyrimidines (FP) alone (i.e., either 5-fluorouracil [5FU] or capecitabine), irinotecan-based regimen (FP-I), or oxaliplatin-based regimen (FP-O). Terminating some agents of chemotherapy was not considered a change in therapy line as long as other agents and biologics in this line continued, and neither was a short data gap (up to a month) of chemotherapy use. The tool enables linking of clinical parameters and treatments to specific outcomes, and can be queried for data analysis. The tool also generates relevant graphics (e.g., Kaplan-Meier plots, histograms, etc) to facilitate interpretation of the data [11, 14].

### Statistical design and analyses

The primary endpoints were OS (defined from the first day of bevacizumab infusion) and duration of treatment (DOT) of first-line bevacizumab-containing regimen. One-way ANOVA was used to compare characteristics between patient subgroups and the Bonferroni method was used to correct for multiple comparisons. The Kaplan Meier method was used to calculate median OS, DOT and 95% confidence intervals (CI). The Cox proportional hazards method was used to assess the effect of various parameters on OS/DOT (adjusted by comorbidities and demographic attributes). The parameters considered included undergoing definitive local therapy such as resection of metastases in the liver or lungs and rarely definitive irradiation of metastatic lymph nodes (referred to hereafter as “Surgery” patients) vs. none (referred to hereafter as “Non-surgery” patients); type of chemotherapy administered and their sequential use; use of anti-EGFR therapy (as second line after bevacizumab-containing regimen); age; gender; comorbidities; regular non-oncology medication use; and laboratory data.

## Results

### Patient population

A total of 753 metastatic CRC patients were included in the analysis (Table 1), of whom 116 patients (15.4%) were in the Surgery group and 637 patients (84.6%) were in the Non-surgery group. Overall, the Surgery group was characterized by being younger (*p* < 0.002), and having a lower proportion of patients with hypertension (*p* = 0.007) (Table 1). In addition, in the Surgery group, use of narcotic drugs was significantly lower (*p* < 0.002).

**Table 1:** Patient characteristics.

	Surgery group			Non-surgery group				
	First line treatment for metastatic disease: FP-O + bev	First line treatment for metastatic disease: FP-I + bev	All patients	First line treatment for metastatic disease: FP-O + bev	First line treatment for metastatic disease: FP-I + bev	First line treatment for metastatic disease: FP + bev	All patients	*p* value (for comparing the Surgery and Non- Surgery groups)
	n = 81	n = 35	n = 116	n = 217	n = 374	n = 46	n = 637	
**Gender, n (%)**								NS
Male	49 (60)	20 (57)	68 (59)	127 (59)	208 (56)	13 (36)	350 (55)	
Female	32 (40)	15 (43)	48 (41)	90 (41)	166 (44)	33 (64)	287 (45)	
**Age, years**								< .002
Median	60	59	60	63	65	77	65	
Mean (SD)	60 (10)	60 (12)	60 (11)	62 (12)	65 (11)	75 (11)	65 (12)	
**Comorbidities, n (%)**								
Diabetes	17 (21)	8 (23)	26 (22)	51 (24)	93 (25)	12 (25)	153 (24)	NS
Hypertension	32 (40)	14 (40)	46 (40)	107 (49)	199 (53)	32 (70)	338 (53)	.007
CVD	13 (16)	4 (11)	20 (17)	38 (18)	56 (15)	11 (23)	108 (17)	NS

FP, fluoropyrimidine; FP-I, FP plus irinotecan; FP-O, FP plus oxaliplatin; bev,bevacizumab; CVD, cardiovascular disease; NS, nonsignificant; SD, standard deviation.

The median follow up for the entire cohort (including Surgery patients) was 18.9 months. The median OS was 20.5 (95% CI, 19.5–23.0) months, and the 5-year survival rate was 15.5%. Treatment patterns differed significantly between the Surgery and Non-surgery groups, with more frequent use of the FP-O 1^st^ line regimen in the Surgery vs. the Non-surgery group (69.8% vs. 34.1%) and less frequent use of the FP-I regimen (30.2% vs. 58.7%) in these respective groups (*p* < 0.001; Table 2). Overall survival and 5-year survival rates also differed significantly between the Surgery and Non-surgery groups (*p* < 0.001; Table 2).

**Table 2:** Treatment patterns and clinical outcomes.

	Surgery group			Non-surgery group			
	First line treatment for metastatic disease: FP-O + bev	First line treatment for metastatic disease: FP-I + bev	All patients	First line treatment for metastatic disease: FP-O + bev	First line treatment for metastatic disease: FP-I + bev	First line treatment for metastatic disease: FP+bev	All patients
	n = 81	n = 35	n = 116	n = 217	n = 374	n = 46	n = 637
**Median duration of bevacizumab treatment, months**^a^	4.9	7.3	6.1	10.3	11.9	9.8	11.4
**Patients proceeding to second line treatment in the metastatic setting, n (%)**	N/A	N/A	N/A	142 (65)	261 (70)	29 (63)	432 (68)
**Clinical outcomes**							
Censored patients, n	55	25	80	44	65	7	116
Median follow-up time, months	27	40	31	17	18	12	17
Median OS (95% CI), months	58.5 (41.6–64.8)	Not reached	Not reached	18.8 (16.6–20.6)	19.2 (17.4–20.5)	12.3 (8.2–18.5)	18.7 (17.2–19.9)
5-year survival rate, %	52	74	59	8	7	7	7

FP, fluoropyrimidine; FP-I, FP plus irinotecan; FP-O, FP plus oxaliplatin; bev, bevacizumab; CI, confidence interval; N/A, not applicable/not available; OS, overall survival; SD, standard deviation.

^a^For the Surgery group, bevacizumab treatment refers to pre-surgery treatment.

### Treatment patterns and clinical outcomes in the surgery group

This group included 116 patients who were followed for a median of 31 months. The median OS for the entire group was not reached. It was 58.5 (95% CI, 41.6–64.8) months for the subgroup of patients treated with FP-O plus bevacizumab and was not reached for patients treated with FP-I plus bevacizumab (Table 2). The median duration of bevacizumab-containing therapy pre-surgery was 6.1 months (patients receiving FP-O, 4.9 months; patients receiving FP-I, 7.3 months). Post-surgery, 93 patients (80.2%) received systemic therapy, of whom 79 (84.9%) resumed therapy within 6 months of surgery (median, 1.9 months). In 44 patients, post-surgery treatment lasted longer than 8 months, suggesting that at least some of these patients may had residual disease or new progression.

The survival rates, and the duration of pre-surgery treatments were not significantly different between Surgery patients treated with FP-O plus bevacizumab and those treated with FP-I plus bevacizumab (Table 2).

### Treatment patterns and clinical outcomes in the non-surgery group

This group included 637 patients who were followed for a median of 17 months. The median OS of the entire Non-surgery group was 18.7 (95% CI, 17.2–19.9) months, and the DOT was 11.4 months. OS and survival rates were similar in patients treated with FP-O plus bevacizumab and those treated with FP-I plus bevacizumab, and were significantly longer compared with patients treated with FP alone plus bevacizumab (*p* < 0.002; Table 2). Patients in the FP group were older (median age of 77 years vs 65 years for the entire Non-surgery group) and likely sicker as indicated by increased likelihood of suffering high blood pressure (Table 1) and by a significantly higher use of antiemetics compared to the two other Non-surgery subgroups (*p* < 0.001).

Cox proportional hazards model demonstrated that the following parameters were statistically significantly associated with inferior OS and DOT (*p* < 0.001 after adjusting for age and gender): baseline elevated parameters for platelet count, leucocyte count, cholesterol, low-density lipoprotein cholesterol, protein in the urine, and the tumor marker CEA. In addition, use of narcotics and corticosteroids was significantly associated with lower OS, and regular use of narcotics, diuretics, and gastrointestinal medications (mostly proton pump inhibitors [PPIs]) was significantly associated with lower DOT (*p* < 0.001 after adjusting for age and gender). Notably, these results remained significant after adjusting for chemotherapy regimen (FP-O, FP-I, and FP).

Of the 637 patients in this group, 339 (53.2%) received both FP-O and FP-I regimens and 85 (13.3%) received only one of these regimens. Patients who received both “aggressive” regimens achieved numerically longer OS compared to those who did not (median [95% CI] of 22.1 [19.9–24.0] vs. 18.9 [15.5–21.9] months); however, the difference was not statistically significant (possibly, due to small sample sizes).

A total of 432 patients received second (or subsequent) lines of therapy. Their median OS was 20.4 (95% CI, 19.5–23) months. Of these patients, 168 (38.9%) received anti-EGFR therapy (142 cetuximab- and 26 panitumumab-containing regimens). To assess the true impact of anti-EGFR therapy, we focused on patients who received a second (or more) lines of therapy in the metastatic setting and that at least one of these additional lines occurred after December 2008, when w.t. KRAS status became mandatory for anti-EGFR therapy (n = 337). Of these patients, 124 with w.t. KRAS were treated with anti-EGFR therapy and their median OS was 25.4 (95% CI, 22.1–29.8) months; and 37 with w.t. KRAS were not treated with anti-EGFR therapy, and their median OS was 18.7 (95% CI, 14.9–39.9) months. OS was numerically longer in the former subgroup although it did not reach statistical significance (possibly, due to small sample sizes).

## Discussion

In this study, we developed a novel computational tool through multi-disciplinary efforts involving computational scientists, oncologists, directors, and pharmacists, and used it to analyze treatment patterns and clinical outcomes in patients with metastatic CRC treated with bevacizumab-containing regimens as first-line therapy. The tool was confirmatory with respect to other well-known factors (e.g., outcomes of patients treated with both FP-O and FP-I were not affected by the sequence of regimens used, anti-EGFR therapy in subsequent lines of therapy benefited, at least numerically, w.t. KRAS patients) and identified non-oncology factors which may adversely impact outcomes such as the use of diuretics or PPIs.

The observation that diuretic use was associated with worse clinical outcomes is consistent with a recent population-based study involving 3,967 CRC patients, showing a statistically significant increase in death risk with thiazide diuretics use [15]. Also, recent studies in gastroesophageal and non-small-cell lung cancer demonstrated that PPI use negatively impacted the efficacy of capecitabine plus oxaliplatin and erlotinib, respectively [16, 17] suggesting that at least for oral drugs (e.g., capecitabine in our study), PPI use may be associated with altered absorption and reduced efficacy.

Our findings (median duration of bevacizumab treatment for the entire Non-Surgery group of 11.4 months, and OS of 20.5 [95% CI, 19.5–23.0] for the entire cohort) are largely consistent with findings from initial phase III RCTs and four phase IV observational studies (progression-free survival [PFS] of 5.4–14 months and OS ranging between 20.3–24.8 months) [6–10, 18–22]. Findings may differ between studies/cohorts according to the chemotherapy backbone used, study time frame, patient selection and comorbidities, and intervals requirements for periodic patient assessment.

Our findings for the subgroup of patients in the Non-surgery group who were treated with bevacizumab plus FP alone (median age, 77 years; median DOT, 9.8 months) are consistent with those reported in the randomized phase III AVEX trial involving 280 elderly metastatic CRC patients (median age, 76 years) treated with this regimen, where the PFS was 9.1 (95% CI, 7.3–11.4) month [23]. Also, our findings for the Surgery group are consistent with those of the phase III EORTC 40983 trial involving 364 patients with resectable liver metastases from CRC where the OS for patients who underwent perioperative oxaliplatin-based treatment (without bevacizumab) was 61.3 months [24].

In our study, FP-O and FP-I were similarly effective as the chemotherapy backbone, consistent with prior reports [18, 25, 26]. Furthermore, receiving both regimens (sequentially) was associated with improved outcomes; however, the sequence of using these regimens did not matter, consistent with previous pre-bevacizumab era studies comparing these sequences [27].

Treatment patterns observed in our study are consistent with those reported in community-based studies including the BRiTE, BEAT, and ARIES with FP-O being used more frequently than FP-I as the chemotherapy backbone [18, 19, 25]. The difference in treatment patterns between Surgery and Non-surgery patients with FP-O more common in the Surgery group and FP-I more common in the Non-surgery group is consistent with treatment guidelines for pre-operative management [28], and the known differences in the profile of hepatic toxicity between the 2 regimens [29].

Our study has several limitations. Analysis of real-life data is limited by the inherently retrospective nature of this analysis and therefore associations with poor outcomes such as those observed for patients using narcotics and corticosteroids may simply reflect poor performance status. Also, some of the subgroups in the analysis are relatively small limiting our ability to draw conclusions (i.e., on cetuximab as 2^nd^ line therapy in w.t. KRAS patients). Furthermore, the current analysis is limited as it does not address safety. The next step in the evolution of this computerized tool will involve inclusion of parameters indicative of adverse events (e.g., hospitalization for supportive care, blood transfusions).

## Conclusions

In this study we describe a novel tool and how its application can provide clinically relevant insights that could facilitate precise patient care. In future, such a tool can be further developed into a “true” machine learning tool that “learns” from a dataset that is prospectively and constantly updated with the accumulation of real-life data and incorporation of modern therapeutic approaches, and enables physicians to make better-informed treatment decisions and learn from the experience of their peers.
